# Lethal effect of heterologous *Haemonchus contortus* α- and β-tubulin expression in *Leishmania tarentolae*

**DOI:** 10.1371/journal.pone.0337417

**Published:** 2026-01-15

**Authors:** Marc Borchert, Jürgen Krücken, Marion Müller, Georg von Samson-Himmelstjerna

**Affiliations:** 1 Institute for Parasitology and Tropical Veterinary Medicine, Freie Universität Berlin, Berlin, Germany; 2 Veterinary Centre for Resistance Research, Freie Universität Berlin, Berlin, Germany; 3 Institute of Immunology, Freie Universität Berlin, Berlin, Germany; Tehran University of Medical Sciences, IRAN, ISLAMIC REPUBLIC OF

## Abstract

Benzimidazoles are widely used anthelmintics that target β-tubulin and disrupt microtubule formation, yet the precise mode of action and resistance mechanisms in parasitic nematodes remains incompletely understood. To investigate the processes, heterologous expression of *Haemonchus contortus* isotype-1 α-tubulin and isotype-1 β-tubulin linked to the selection markers nourseothricin and hygromycin, respectively, was attempted following integration into *Leishmania tarentolae ssu(18S)* loci. Transcription of both tubulin genes was confirmed by RT-PCR two days post-transfection. However, transgenic *L. tarentolae* failed to recover or enter exponential growth under antibiotic selection. In contrast, control constructs containing scrambled open reading frame integrated successfully and yielded viable and proliferating lines expressing the corresponding mRNAs. These findings indicate that translation of functional *H. contortus* tubulins is toxic to *L. tarentolae*, likely due to incompatibility with native tubulins and disruption of the native microtubule network, resulting in loss of motility and cell death. The study provides experimental evidence that cross-species tubulin expression can be lethal due to cytoskeletal interference, underscoring the functional specificity of tubulin subunits. This incompatibility represents a barrier to heterologous tubulin expression and suggests that controlled or inducible eukaryotic expression systems may be necessary for the functional production of nematode tubulins.

## Introduction

Parasitic nematodes, particularly *Haemonchus contortus*, cause severe diseases and production losses in livestock, resulting in considerable socioeconomic damage [1]. The parasite *H. contortus* infects sheep, goats, and cattle, posing a severe threat to animal health [2]. Furthermore, *H. contortus* is known for its rapid development of resistance to all classes of anthelmintics, including benzimidazoles (BZs) [1,3,4]. Effective against many helminths in mammals BZs are broad-spectrum anthelmintics [5,6]. By selectively binding to isotype-1 β-tubulin (TBB-1) of parasitic nematodes, BZs inhibit the polymerization of tubulin heterodimers into microtubules [7,8].

Polymorphisms such as Q134H [9], F167Y [10], E198A [11], E198L [12], and F200Y [13] in TBB-1 confer resistance to BZs in parasitic nematodes. To improve the understanding of the mode of action and mechanism of resistance, it is important to characterize and structurally analyze parasite tubulins. However, previous attempts to recombinantly express *H. contortus* isotype-1 α-tubulin (*Hco*TBA-1) and isotype-1 β-tubulin (*Hco*TBB-1) have faced major limitations. In *Escherichia coli*, these tubulins were successfully expressed and purified, but their functionality was not verified, raising concerns about proper folding due to the absence of eukaryotic folding machinery, including the TRiC/CCT complex, a factor essential for correct tubulin folding, and its absence often causes misfolded or non-functional proteins [14]. In *Saccharomyces cerevisiae*, the essential role of endogenous β-tubulin poses significant challenges for heterologous expression, as *Hco*TBB-1 was unable to substitute the endogenous yeast β-tubulin, and its expression proved lethal [15].

These limitations highlight the need for an alternative system capable of expressing functional recombinant tubulin with the appropriate folding and posttranslational modifications. Currently, only an *in silico* modeling analysis of albendazole bound to *Hco*TBA-1/*Hco*TBB-1 has been conducted, providing valuable insight into the molecular basis of resistance. Although the model suggests a plausible binding mode, it has not yet been experimentally confirmed [16]. This study aimed to recombinantly express *Hco*TBA-1 and *Hco*TBB-1 in the eukaryotic expression system *Leishmania tarentolae* (LEXSY), providing an alternative system for heterologous protein expression to traditional yeast [17,18], insect (e.g., Sf-9 cells) [19,20], and mammalian cell systems (e.g., HEK293 cells) [21]. This system provides robust growth, cost-efficiency, and enables complete eukaryotic protein folding machinery [22]. Further, LEXSY offers a broad range of post-translational modifications (PTMs), including mammalian-like glycosylation [23], phosphorylation, acetylation [24], polyglutamylation, and detyrosination/tyrosination [25]. Although polyamination remains uncharacterized and polyglycylation is absent [26], LEXSY provides a safe and flexible system for recombinant protein expression, with a wide range of PTMs, higher yields, and faster growth compared to other eukaryotic systems [27].

## Materials and methods

### Maintenance and cultivation of LEXSY P10 promastigotes

The cultivation of the promastigote stage of *L. tarentolae* (LEXSY strain P10, Jena Bioscience, Jena, Germany) was conducted in ventilated T-25 and T-75 cell culture flasks (Sarstedt, Nürnbrecht, Germany) in BHI medium (Brain Heart Infusion medium) supplemented with 5 µg/mL porcine Hemin (Jena Bioscience, Jena, Germany), 2.5 U/mL penicillin (base), and 250 µg/mL streptomycin (base) (Jena Bioscience, Jena, Germany) under aerobic conditions at 26 °C. For strain maintenance, the *Leishmania* culture was diluted 1:20 in freshly supplemented BHI medium during the log phase (OD_600nm_ of 2–3).

### Codon optimization and cloning of *Haemonchus contortus* tubulin genes into expression plasmids

**Fig 1:**
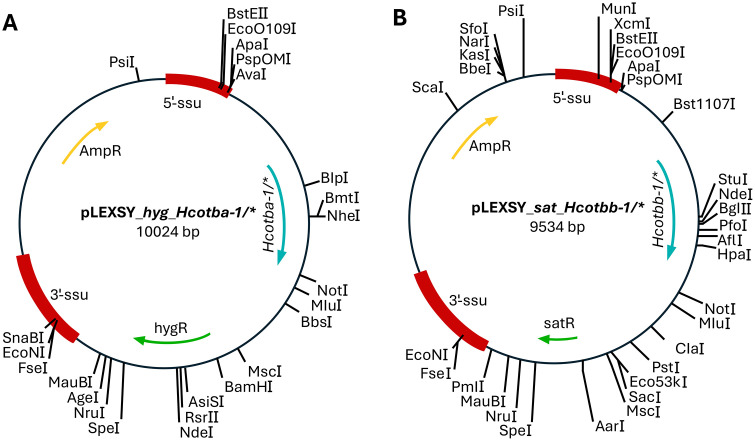
Plasmid maps of the cloned constructs generated using the HiFi DNA Assembly Kit. **A)** The Hcotba-1 cDNAs, either with an intact ORF or a scrambled ORF, were inserted into the pLEXSY_hyg^R^ vector backbone. **B)** Similarly, the Hcotbb-1 cDNAs, with an intact ORF or a scrambled ORF, were inserted into the pLEXSY_sat^R^ vector backbone. These plasmids do not contain functional promoters for L. tarentolae.

The cDNA constructs of *tba-1* and *tbb-1* from *Haemonchus contortus* (GenBank: L02108.1 and EF198865.1) were designed according to the protocol described by Ti et al. (2020). For codon optimization, the online tool OPTIMIZER (http://genomes.urv.es/OPTIMIZER/) was used in combination with the codon usage table for *L. tarentolae*, obtained from the Codon Usage Database (http://www.kazusa.or.jp/codon/). Two codon optimization approaches were applied: one using the standard genetic code, resulting in constructs with an intact open reading frame (ORF) (*tba-1* and *tbb-1* codon opt.), and another based on mold, protozoan, and coelenterate mitochondrial genetic code, leading to constructs with a scrambled ORF (*tba-1* codon opt.* and *tbb-1* codon opt.*) (S1 Table in S1 File). The cDNAs, with complementary ends (S2 Table in S1 File), were cloned into *Bgl*II (Thermo Fisher Scientific, Waltham, MA, USA) and *Kpn*I (Thermo Fisher Scientific, Waltham, MA, USA) linearized backbone plasmids (pLEXSY_*sat*^*R*^ and pLEXSY_*hyg*^*R*^, Jena Bioscience, Jena, Germany) using the highly efficient HiFi DNA Assembly method (New England Biolabs, Ipswich, MA, USA) (Fig 1). These plasmids do not contain a functional promoter for *L. tarentolae*. To confirm the successful integration of the cDNA into the backbone plasmids, Sanger sequencing was performed at LGC Genomics (Berlin, Germany).

### Preparation of linearized expression cassettes for transfection

A total amount of 10 µg expression plasmid in 50 µL was linearized by digestion with 0.4 U/µL *Smi*I (*Swa*I) (Thermo Fischer Scientific, Waltham, MA, USA) overnight at 30 °C. To ensure optimal transfection efficiency, the expression cassette containing the target gene was isolated from the gel using the Gel DNA Recovery Kit (Zymo Research, Freiburg, Germany). The linearized expression cassette was cut out from the gel and pooled on a Zymo-Spin column. The remaining gel isolation steps were conducted according to the manufacturer’s protocol. In the end, the linearized expression cassette was eluted in 10 µL of DEPC water.

### Transfection of LEXSY host P10 with linearized expression cassettes by Amaxa electroporation (nucleofection)

For constitutive expression, the LEXSY host P10 was transfected using linearized expression cassettes: pLEXSY_*sat*^*R*^_*Hcotba-1* and pLEXSY_*hyg*^*R*^_*Hcotbb-1*, both containing intact ORFs. As a control, transfections were performed with the linearized expression cassettes pLEXSY_*sat*^*R*^_Hcotba-1* and pLEXSY_*hyg*^*R*^_Hcotbb-1*, which contained scrambled ORFs. All transfections were conducted via Amaxa electroporation. For each construct (intact ORF, scrambled ORF, and mock transfection), three biological replicates were performed. Initially, a pre-culture of the LEXSY host was inoculated in a 1:20 ratio in 10 mL of BHI^+^ medium (supplemented with porcine Hemin and Penicillin/streptomycin) and incubated for 3 days at 26 °C. After 3 days, the pre-culture was diluted 1:10 in 10 mL of BHI^+^ medium and grown to an OD_600nm_ of 1.2 to 1.4 (6 × 10^7^ cells per mL). The cells were then transferred to a 15 mL centrifugation tube and chilled on ice for 10 min, followed by centrifugation at 2,000 × g for 5 min at 4°C. After discarding 5 mL of the supernatant, the cells were gently resuspended in the remaining 5 mL and returned to ice for an additional 5 min. For electroporation, 1 mL of the cells was transferred to a 1.5 mL reaction tube and pelleted by centrifugation at 2,000 × g for 5 min at RT. The supernatant was carefully removed, and the pellet was resuspended in 100 µL of Nucleofector reagent (82 µL Nucleofector Solution + 18 µL Supplement) (Human T Cell Nucleofector Kit; Lonza, Visp, Schweiz) and placed on ice. Meanwhile, 1.5 µg of each linearized expression cassette, dissolved in a maximum volume of 10 µL ddH_2_O, was added to the Amaxa electroporation cuvette on ice. Next, 100 µL of chilled cells were carefully transferred into the electroporation cuvette, ensuring that no air bubbles were present. The Amaxa Nucleofector II system was used for the electroporation with program U33. Following the electroporation, the cuvettes were placed on ice for 5 min. The cells were then transferred to 10 mL of pre-warmed BHI^+^ medium, and the cuvette was rinsed with 300 µL of pre-warmed BHI^+^ medium to maximize cell recovery. The culture was incubated at 26 °C until an OD_600nm_ of 0.3 to 0.4 was reached. Once this optical density range was detected, Nourseothricin (Jena Bioscience, Jena, Germany) and Hygromycin B (Carl Roth, Karlsruhe, Germany) were added to the culture to obtain a final concentration of 100 µg/mL for both. Thereafter, the OD_600nm_ was measured at the same time each day.

### Isolation of genomic DNA from LEXSY promastigotes

Genomic DNA from LEXSY promastigotes was purified using the NucleoSpin Tissue Kit (Macherey-Nagel, Düren, Germany) after growing the culture to an OD_600nm_ of 1.2 to 1.4 (6 × 10^7^ cells per mL). Afterward, 200 µL of the culture was centrifuged at 16,000 × g. Next, 25 μL of 23 mg/mL Proteinase K solution and 200 μL of B3 buffer were added, followed by a 15 min incubation step at 70 °C. The remaining steps for genomic DNA purification were conducted according to the “Standard protocol for human or animal tissue and cultured cells” protocol (Macherey Nagel, Düren, Germany).

### Isolation of RNA from LEXSY promastigotes

RNA from LEXSY promastigotes was purified using the NucleoSpin RNA Kit (Macherey-Nagel, Düren, Germany), with the culture grown to an OD_600nm_ of 1.2 to 1.4 (6 × 10^7^ cells per mL) before extraction. Then 100 µL of the culture was centrifuged at 16,000 × g, and the resulting pellet was resuspended in 350 µL of RA1 buffer. After resuspension, 3.5 µL 200 µM DTT was added. The remaining steps for RNA purification were conducted according to the “RNA purification from cultured cells and tissue” protocol.

### First strand cDNA synthesis

First strand cDNA synthesis was conducted using a combination of random hexamer oligonucleotides and oligo(dT)_18_ primers with Maxima H-Minus Reverse Transcriptase (Thermo Fisher Scientific, Waltham, MA, USA). The reaction mixture contained 100 pmol of random hexamers, 100 pmol of oligo(dT)_18_ primers, 0.5 mM dNTPs (final concentration in the 20 µL reaction volume), and 13 µL of DNase-treated isolated RNA in a total volume of 14.5 µL. This mixture was incubated at 65 °C for 5 min. Afterwards, 1 × RT buffer, 1 U/µL RiboLock RNase Inhibitor, and 10 U/µL Maxima H Minus Reverse Transcriptase were added in a total volume of 20 µL. The reaction was incubated at 25 °C for 10 min, followed by 50 °C for 15 min, and then terminated by heating at 85 °C for 5 min. The synthesized cDNA was either used immediately for conventional PCR or stored at −20 °C.

### Conventional PCRs for confirmation of genomic integration

**Fig 2:**
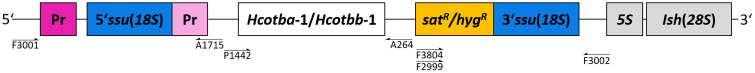
Chromosomal 18S rRNA locus (ssu(18S)) after the integration of the Hcotba-1 and Hcotbb-1 genes into the expression site. Transcription of the Hcotba-1 and Hcotbb-1 genes is driven by the strong RNA polymerase I, regulated by a chromosomal ribosomal promotor (Pr) [28]. The 5’-ssu(18S) and 3’-ssu(18S) are homology arms for homologous recombination. The selection markers sat^R^ and hyg^R^ confer resistance to nourseothricin and hygromycin **B.** The arrows indicate the positions of the forward and reverse primers used in the conventional PCRs.

To verify the integration of the linearized expression cassettes pLEXSY_*sat*^*R*^_Hcotba-1* and pLEXSY_*hyg*^*R*^_Hcotbb-1*, both containing scrambled ORFs into the *ssu(18S)* loci, four conventional PCRs were conducted (Fig 2). The following PCR conditions were used for all conventional PCR reactions: 0.5 µM of both forward and reverse primers, 200 µM dNTPs, 1 × HF buffer, 50 ng genomic DNA, and 0.02 U/µL S7 Fusion Polymerase (Biozym, Hessisch Oldendorf, Germany) in a total volume of 20 µL. The thermocycling conditions included an initial denaturation step at 98 °C for 30 s, followed by 35 cycles of denaturation at 98 °C for 5 s, annealing at 53 or 60 °C for 30 s, and elongation at 72 °C for 40 s, with a final elongation at 72 °C for 5 min. For the first conventional PCR, integration at the left homology arm of the *ssu(18S)* locus was verified using the 5’-*ssu(18S)* forward primer F3001 and the 5’-*ssu(18S)* reverse primer A1715, with an annealing temperature of 60 °C (PCR product size: 1,027 bp) (S2 Table in S1 File). The second conventional PCR amplified the target genes Hcotba-1* and Hcotbb-1*, using primers P1442 and A264, with an annealing temperature of 60 °C (PCR product size for Hcotba-1*: 1,631 bp; PCR product size for Hcotbb-1*: 1,526 bp) (S2 Table in S1 File). To assess the integration of the selection markers *sat*^*R*^ and *hyg*^*R*^, conferring nourseothricin and hygromycin resistance at the right homology arm of the *ssu(18S)* locus, the 3’-*ssu(18S)* forward primers A3804 or D2999 and the 3’-*ssu(18S)* reverse primer F3002 were used, respectively, with an annealing temperature of 53 °C (PCR product size for *sat*^*R*^: 1,823 bp; PCR product size for *hyg*^*R*^: 2,771 bp). All PCR products were visualized by electrophoresis on a 1.5% (w/v) agarose gel, stained with 1 × Ultra DNA Gel Stain (ABP Biosciences, Rockville, MD, USA). PCR products were excised and purified from agarose gels using the Zymoclean Gel DNA Recovery Kit (Freiburg, Germany), cloned into the StrataClone Blunt Vector pSC-B-amp/kan (Agilent Technologies, La Jolla, CA, USA), and sequenced at LGC Genomics (Berlin, Germany).

### Evaluation of *Hco*tbb-1 gene expression by conventional PCR

To detect the gene expression of *Hcotbb-1* with an intact ORF and Hcotbb-1* with a scrambled ORF, cDNAs were synthesized from the RNA isolated from transgenic strains. These synthesized cDNAs were used as templates in conventional PCR to amplify the specific target sequences. The following conditions were applied for all conventional PCRs: 0.5 µM of both forward and reverse primers (S2 Table in S1 File), 200 µM dNTPs, 1 × HF buffer, 2 µL cDNA, and 0.02 U/µL S7 Fusion Polymerase (Biozym, Hessisch Oldendorf, Germany) in a total volume of 50 µL. The thermocycler parameters consisted of an initial denaturation at 98 °C for 30 s, followed by 35 cycles of denaturation at 98 °C for 10 s, annealing at 61 °C for 30 s, and elongation at 72 °C for 30 s, with a final elongation step at 72 °C for 10 min. All PCR products were visualized by electrophoresis in 1.5% (w/v) agarose gels stained with 1 × Ultra DNA Gel Stain (ABP Biosciences; Rockville, MD, USA). Following gel electrophoresis, PCR products were excised and purified using the Zymoclean Gel DNA Recovery Kit (Freiburg, Germany), cloned into the StrataClone Blunt Vector pSC-B-amp/kan (Agilent Technologies, La Jolla, CA, USA), and sequenced by Sanger sequencing at LGC Genomics (Berlin, Germany).

### Statistical analysis

Statistical analyses were conducted using R v4.2.1 or GraphPad Prism v10.03 for Windows (GraphPad Software, San Diego, California, USA, www.graphpad.com). For determining the R² value and the doubling time from the nonlinear regression exponential growth curves, GraphPad Prism was used. Specifically, the *Exponential (Malthusian) growth* model was applied as *Y = Y*_*0*_*· exp(k· X),* where *Y*_*0*_ represents the starting population, *k* the rate constant (inverse of the X time units), and the *doubling time* corresponds to ln(2)/k. Subsequently, the x and y values along with the nonlinear fit were visualized in R. This was achieved by creating an XY plot using the *ggplot2* (v 4.0.0) package and performing the nonlinear regression with the *fit_exponential* function. Further, to assess differences between the three groups (scrambled ORF, intact ORF, and mock transfection), a Kruskal–Wallis test was performed separately for each time point. When the Kruskal–Wallis test indicated a statistically significant difference (p < 0.05), post-hoc pairwise comparisons were conducted using Dunn’s test with Holm correction for multiple testing. Analyses were carried out using the R packages *FSA* (v. 0.10.0), *tidyr* (v.1.3.1), and *dplyr* (v. 1.1.4). OD_600nm_ values below the detection limit of 0.01 were replaced with 0.0099 prior to analysis to allow statistical processing.

## Results

### Growth kinetics during polyclonal selection of transgenic LEXSY P10 promastigotes

**Fig 3:**
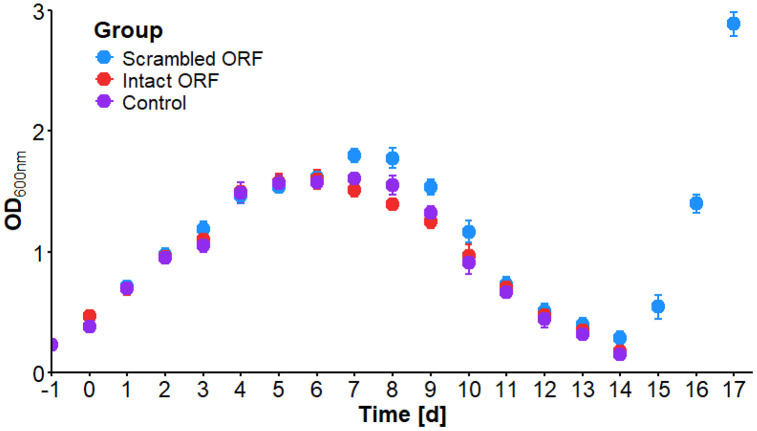
Selection curves of transgenic LEXSY P10 after Amaxa electroporation in BHI^+^ medium. Each group consisted of three replicates. The intact ORF group was able to express *Hco*TBA-1 and *Hco*TBB-1, the scrambled ORF group was unable to express *Hco*TBA-1 and *Hco*TBB-1, and the control group was a mock transfection without DNA.

To investigate the effects of different ORF constructs on cell growth, cells were transfected with either the linearized expression cassettes: pLEXSY_*hyg*^*R*^_*Hcotba-1* and pLEXSY_*sat*^*R*^_*Hcotbb-1* (intact ORF), pLEXSY_*hyg*^*R*^_Hcotba-1* and pLEXSY_*sat*^*R*^_Hcotbb-1* (scrambled ORF), or a mock transfection control without plasmids. For each expression cassette or control, three biological replicates were performed. Following transfection, LEXSY P10 were cultured in pre-warmed BHI^+^ medium at 26 °C until an OD_600nm_ of 0.3 to 0.4 was reached. The transgenic LEXSY P10 promastigotes were then directly selected in BHI^+^ medium for 17 days (S3 Table in S1 File). The resulting selection curves can be divided into three key phases. The first phase, the lag phase, occurred from day 0 to day 4, during which the cells grew and adapted to the antibiotics. During this phase, the doubling time was 2.13 days (scrambled ORF; not expressing tubulins), 2.28 days (intact ORF; expressing tubulins), and 2.22 days (mock transfection) (Fig 3, S1A Fig in S1 File). A Kruskal–Wallis test (p > 0.05) revealed no statistically significant differences between the groups. The second phase, the death phase, occurred from day 9 to day 14. During this phase, the death of non-transfected cells began, while the transfected resistant cells started to proliferate. This led to an exponential decrease in cell numbers, with the OD_600nm_ halving every 1.95 days (scrambled ORF; not expressing tubulin), 2.21 days (intact ORF; expressing tubulin), and 1.94 days (mock transfection) (Fig 3, S1B Fig in S1 File). Again, no statistically significant differences were observed between the groups (Kruskal–Wallis test, p > 0.05). The final phase, the exponential growth phase, was marked by the proliferation of only the transfected, resistant cells, resulting in an increase in cell population. This phase had a doubling time of 0.89 days (scrambled ORF; not expressing tubulin) (Fig 3). A Kruskal–Wallis test revealed no statistically significant differences between the groups on day 15 (p > 0.05), whereas significant differences were observed on days 16–17 (p < 0.05). Post-hoc pairwise comparisons using Dunn’s test indicated that on days 16–17, the scrambled ORF group differed significantly from both the intact ORF and mock transfection groups, while no significant differences were detected between the intact ORF and mock transfection groups. However, exponential growth was observed only in the group that expressed the selection markers, but not *Hco*TBA-1 and *Hco*TBB-1. In contrast, the group that expressed the selection markers along with *Hco*TBA-1 and *Hco*TBB-1 did not undergo exponential growth, neither did the mock transfected group (Fig 3). Despite the expression of the selection markers, the *Leishmania* group with the intact ORF of *Hcotba-1 and Hcotbb-1* showed no active movement through flagellar propulsion after just one day and instead only exhibited twitching, followed by a complete loss of motility within the next day.

### Confirmation of *Hcotba-1* and *Hcotbb-1* genomic integration

To verify the integration of the Hcotba-1* and Hcotbb-1*, both with scrambled ORF cassettes into the *ssu*(*18S)* loci, four conventional PCRs were conducted. In the first PCR, the primer pair F3001 and A1715 flanking the left homology arm (5’-*ssu*(*18S*)), including the *L. donovani* rRNA promoter fragment (Fig 2), was used. This PCR confirmed the successful integration of the expression cassettes at the left homology arm, amplifying an amplicon of 1,027 bp (S2A Fig in S1 File). The second PCR, using the primer pair P1442 and A264, amplified Hcotba-1* (1,631 bp) and Hcotbb-1* (1,526 bp) (S2B Fig in S1 File). However, electrophoresis was unable to distinguish between the two amplicons. These amplicons were subsequently cloned and Sanger sequenced, which confirmed the presence of both tubulin transgenes. Lastly, the selection markers *sat*^*R*^ (1,823 bp) and *hph*^*R*^ (2,771 bp) were amplified using forward primers A3804 or D2999, which hybridize within *sat*^*R*^ and *hph*^*R*^, respectively, and the reverse primer F3002, which binds downstream of the right homology arm (Fig 2, S2C Fig in S1 File).

### Verification of *Hcotbb-1* gene expression in transgenic LEXSY strains

To verify the gene expression of *Hcotbb-1* with an intact ORF and Hcotbb-1* with a scrambled ORF, the RNAs isolated from the three LEXSY strains were reverse transcribed into cDNA using M-MuLV RT. For this purpose, RNA was isolated from the three LEXSY strains at the same time each day, with the cell count determined by measuring the OD_600nm_ to ensure consistent RNA isolation from the same number of cells. After the cDNA synthesis, the cDNA was used as a template for conventional PCR. The first LEXSY strain, which was mock transfected, did not show any amplicon at 1,315 bp (S3A Fig in S1 File). The second LEXSY strain, transfected with Hcotba-1* and Hcotbb-1*, showed an amplicon at 1,315 bp from day 1 to day 13 (S3B Fig in S1 File). This proves that the mRNA with the scrambled open reading frame was expressed throughout the observation period. The third LEXSY strain, transfected with *Hcotba-1* and *Hcotbb-1*, exhibited an amplicon at 1,191 bp at day 1 and day 2 (S3C Fig in S1 File). This indicates that cells expressing the mRNA with the translatable *Hcotbb-1* were lost from the cultures after day 2.

## Discussion

In a parallel experimental setup, constructs with intact and scrambled ORFs were tested alongside mock transfection (water) controls. This parallel approach ensured that the effects of expression of the *H. contortus* tubulin on *Leishmania* cell viability could be directly compared under identical conditions. When the standard genetic code was applied, *Hcotba-1* and *Hcotbb-1* were presumably translated correctly. However, this impaired *L. tarentolae* recovery post-transfection and prohibited exponential growth, suggesting that *H. contortus* tubulin expression was toxic to the *Leishmania* parasites. In contrast, when scrambled ORFs were used, translation of the recombinant tubulins growth were not possible in *L. tarentolae*, indicating that the observed toxicity was associated explicitly with functional tubulin expression rather than the presence of the constructs themselves or the nucleofection process. The assumption that the recombinant tubulin expression is toxic to *L. tarentolae* is further supported by the lack of proliferation observed in the group expressing recombinant tubulins compared to the group not expressing recombinant tubulins. In the group able to express recombinant *H. contortus* tubulins, the *Leishmania* were unable to recover. In contrast, the group with an integrated expression cassette but unable to translate the recombinant tubulins showed exponential proliferation, despite drug selection starting from day 14. This observation indicates a lethal phenotype in the group potentially expressing recombinant nematode tubulins. The presence of recombinant nematode tubulins might disrupt essential cellular processes in the *L. tarentolae*.

To confirm correct integration in lines transfected with scrambled ORF, conventional PCRs spanning the homology arms were conducted. The results confirmed the successful integration of both constructs unable to express α- and β-tubulin into the *ssu(18S)* loci. However, intact ORF constructs could not be confirmed by conventional PCRs, as no living *Leishmania* remained after 14 days for DNA isolation. Therefore, RT-PCRs were conducted to confirm the transcription of the *H. contortus* β-tubulin transgenes from day 1 to day 13 after selection, providing direct evidence of transcription and thus successful integration. Transcription of Hcotbb-1* was detectable throughout the observation period, while *Hcotbb-1* transcription was only present on days 1 and 2. This most likely supports the integration of scrambled ORF constructs downstream of a functional promoter and provides indirect evidence for intact ORF constructs followed by early loss of transgenic cells. Since the transfected plasmids did not contain functional promoters, these results imply that integration into *ssu*(*18S*) loci (or downstream of other sites that could function as promoter) occurred, but translation of *Hcotbb-1* was apparently lethal to *L. tarentolae.*

The most likely cause of lethality is the disruption of the cytoskeleton, which is critical for *Leishmania* spp. as well as all eukaryotic cells [29–31]. Tubulin heterodimers are integral components of microtubules (MTs) that are essential for mitosis, flagellar function, and endocytosis. Incorporation of recombinant tubulin likely disturbed the native MT network involved in cytoskeletal stability, cell cycle regulation, and motility. Indeed, flagellar function appeared disrupted, as *L. tarentolae* showed only twitching and rapidly lost motility. Although protein expression levels were not quantified, the rapid motility defects and disappearance of transgenic populations suggest toxicity due to recombinant tubulin expression, which may have also inhibited cell division by impairing the cytoskeleton.

Beyond the toxicity resulting from recombinant tubulin expression, subunit incompatibility between *Hco*TBA-1 and *Hco*TBB-1 may have further contributed to the observed phenotype. *Hco*TBA-1 was selected as the α-tubulin subunit based on its high sequence identity to *C. elegans* α-tubulin (*Cel*TBA-1), which interacts with *C. elegans* β-tubulin (*Cel*BEN-1). Both *Cel*TBA-1 and *Cel*BEN-1 have been implicated in key developmental processes such as neuronal pathfinding [32,33]. However, the functional compatibility between heterologous α- and β-tubulin subunits remains a topic of debate, as different studies have reported varying results depending on the substitution model and experimental context [34,35]. However, *H. contortus* expresses multiple α-tubulin isoforms, and the interaction partner of *Hco*TBB-1 remains unknown. It is therefore possible that another isoform, such as *Hco*TBA-2, is the native heterodimer partner. If so, co-expression of *Hco*TBA-1 and *Hco*TBB-1 may have led to improper dimerization, misfolding, degradation, or toxic accumulation due to incompatibility. This could further destabilize the cytoskeleton and exacerbate the lethal phenotype observed.

Regarding recombinant tubulin expression systems, prokaryotic systems were excluded due to a lack of the chaperonin TRiC/CCT complex required for tubulin folding and the absence of necessary post-translational modifications (PTMs), leading to non-functional proteins or insoluble aggregates [36–39]. In contrast, eukaryotic systems, including baker’s yeast, insect (*Sf*-9) and mammalian cells, possess the TRiC/CCT complex, enabling correct tubulin folding [40]. However, the baker’s yeast expression system is limited in PTMs, making functional expression of tubulin challenging [41–43]. In contrast, mammalian expression systems such as HEK293 cells offer a broad spectrum of PTMs and enable tubulin production with native-like structural and functional properties, although they are associated with high complexity and costs [44]. Insect cell based expression systems, such as *Sf-*9 cells, represent an intermediate solution, providing relatively high protein yields and the ability to perform several eukaryotic PTMs [20,45]. Recombinant human tubulin has been successfully expressed in *Sf-9* cells in at least three independent studies, highlighting the general suitability of this system for producing complex cytoskeletal proteins [20,45,46]. However, functional expression of *H. contortus* or any other nematode’s tubulin in *Sf*-9 cells has not yet been demonstrated. Thus, while this system offers a promising potential, its applicability for the expression of biologically active nematode tubulin remains to be experimentally confirmed and should be considered a prospective direction for further investigations [41–43].

## Conclusions

The *H. contortus* tubulin expression constructs were successfully integrated into the *L. tarentolae ssu(18S)* loci but could not be functionally expressed, likely due to their toxicity, which disrupted essential cellular structures such as the cytoskeleton and flagellum. While prokaryotic expression systems lack the necessary tubulin folding machinery and PTMs, Sf-9 insect cells offer a promising eukaryotic alternative, having successfully produced recombinant tubulin in previous studies. However, their suitability for *H. contortus* tubulin is yet to be experimentally validated and should be explored in future work.

## Supporting information

S1 File Supplemental figures and tables. (PDF)
