# Supplementary material for: Lethal effect of heterologous Haemonchus contortus α- and β-tubulin expression in Leishmania tarentolae
Source: PLoS One. 2026 Jan 15;21(1):e0337417. doi: 10.1371/journal.pone.0337417 (PMC12806841; doi:10.1371/journal.pone.0337417)
Supplement: S1 File — (PDF) [file pone.0337417.s001.pdf]

Supporting information:

S1 Table. Synthesized gBlock Gene Fragments by Integrated DNA Technologies. His$_{10}$ tag; Strep tag II, TEV cleavage site; AP linker; GS linker

gBlocks	Sequence (5‘->3‘)
Hcotba-1 N-His$_{10}$ tag	ATG**CATCATCACCATCACCATCATCACCACCACGAAAACCTGTACTTCCAGGG**<br>**TGCCCCG**ATGCGAGAGGTTATTTCTATTCATATCGGTCAGGCGGGCGTTCAGA<br>TCGGCAACGCATGCTGGGAACTCTATTGCTTGGAGCACGGCATTCAGCCAGAC<br>GGACAGATGCCGAGTGATAAGAGCCTCGGCGGGTGCGACGACTCTTTCTCTAC<br>CTTCTTTAGCGAAACTGGAAGCGGACGTCATGTACCAAGAGCCGTGATGATTG<br>ACCTGGAGCCCACCGTGATCGACGAGATCCGAACTGGGACTTATCGTAGCTTG<br>TTTCACCCTGAGCAGTTGATCACGGGTAAGGAGGACGCGGCAAACAACTACGC<br>GCGTGGCCACTACACCATTGGCAAAGAGATCATTGATCTGACCCTGGACCGCA<br>TTCGGCGACTTGCGGATAATTGCACGGGCCTCCAAGGCTTCCTCGTGTTCCAC<br>TCTTTTGGCGGTGGCACTGGCAGTGGATTCACCTCGTTGCTCATGGAGCGCCT<br>TTCTGTGGACTACGGCAAGAAAGCTAAGCTCGAGTTCTCCGTTTACCCGGCCC<br>CCCAGGTGAGCACAGCCGTTGTTGAGCCATACAATAGCATCCTTACAACCCAT<br>ACGACACTCGAGCATAGTGACTGCAGCTTCATGGTGGATAACGAGGCGATCTA<br>CGATATCTGCCGCCGTAACCTGGACATCGAGCGCCCTAGCTACACAAACCTTA<br>ACCGGCTGATTGGCCAGATCGTCTCTTCCATCACGGCGTCACTGCGCTTTGAC<br>GGCGCCCTGAACGTTGACCTGACCGAGTTCCAGACGAATCTCGTGCCTTACCC<br>GAGGATTCACTTTCCACTCGCGACCTTCAGCCCAGTGATTAGCGCCGAAAAGG<br>CCTACCACGAGCAGCTATCCGTGGCGGAGATCACGAACATGTGCTTCGAGCCG<br>CACAACCAGATGGTTAAGTGCGACCCACGCCACGGCAAATACATGGCGGTCTG<br>TCTCCTCTTCAGGGGTGATGTGGTACCCAAGGACGTCAATGCGGCAATCGCGA<br>CGATTAAGACGAAGCGCAGCATTCAGTTCGTGGACTGGTGCCCGACTGGTTTC<br>AAGGTCGGCATCAACTACCAGCCGCCTACCGTCGTGCCCGGGGGGGACCTAGC<br>CAAGGTACCACGCGCGGTGTGCATGCTCTCGAACACGACCGCGATCGCGGAGG<br>CCTGGGCGCGTCTGGACCACAAGTTCGATCTCATGTACGCGAAGCGCGCTTTC<br>GTCCATTGGTACGTAGGCGAGGGTATGGAGGAGGGCGAGTTCTCTGAGGCACG<br>GGAAGACCTCGCGGCCCTGGAAAAAGACTACGAGGAGGTGGGCGTGGACTCAC<br>TGGAGGACAATGGCGAGGAGGGGGATGAGTATTAA
*Hcotba-1** N-His$_{10}$ tag	ATG**CACCACCATCATCATCATCACCATCATCACGAAAACTTATATTTTCAGGG**<br>**G**GCGCCAATGCGGGAAGTAATCAGCATTCACATAGGTCAGGCGGGTGTTCAAA<br>TTGGCAACGCTTGTTGAGAATTATATTGTTTAGAACATGGTATACAACCAGAT<br>GGGCAAATGCCGTCTGATAAGTCATTAGGCGGGTGTGATGACTCATTTAGCAC<br>TTTTTTTAGTGAAACGGGTTCAGGTCGTCATGTGCCTCGTGCAGTAATGATCG<br>ATTTAGAACCCACGGTGATTGATGAAATTCGCACTGGTACATATAGGTCATTA<br>TTTCACCCTGAACAATTAATTACAGGGAAAGAAGATGCCGCAAACAATTATGC<br>GAGGGGGCATTATACAATTGGAAAAGAAATAATAGATTTAACACTTGACCGCA<br>TACGTCGCTTAGCAGATAATTGCACAGGTCTGCAGGGATTTTTGGTGTTTCAC<br>TCTTTTGGCGGAGGTACGGGTAGTGGATTTACCAGTTTATTAATGGAAAGATT<br>ATCAGTGGATTATGGAAAGAAGGCAAAGTTAGAGTTTTCGGTGTATCCAGCGC<br>CACAGGTAAGTACTGCAGTTGTAGAGCCCTATAATAGTATATTAACTACGCAT<br>ACAACATTAGAACATTCTGATTGTTCATTTATGGTGGACAACGAAGCCATTTA<br>TGATATATGTCGGCGTAACTTAGACATAGAGCGACCATCTTATACAAACTTAA<br>ACAGATTAATTGGTCAAATAGTAAGTAGTATTACTGCTAGCTTACGATTTGAC<br>GGTGCGTTAAACGTAGATTTAACAGAATTTCAGACCAACTTAGTGCCTTATCC<br>ACGTATCCATTTTCCTTTAGCTACTTTTTCACCTGTTATTTCTGCGGAAAAAG<br>CATATCATGAACAACTTTCTGTGGCAGAGATAACAAACATGTGCTTTGAGCCA<br>CATAACCAAATGGTGAAGTGTGATCCCCGACACGGTAAATATATGGCGGTCTG<br>TTTGTTATTTCGAGGTGACGTGGTTCCGAAGGATGTCAATGCAGCCATAGCCA<br>CTATTAAGACAAAGCGTAGTATACAATTTGTTGACTGGTGTCCAACAGGGTTT<br>AAAGTTGGTATTAACTATCAGCCGCCCACGGTAGTGCCAGGAGGTGATTTAGC<br>TAAGGTTCCACGTGCAGTTTGCATGTTAAGCAATACTACGGCCATAGCCGAAG<br>CGTGAGCTCGATTAGATCACAAATTTGATTTAATGTATGCCAAGCGCGCGTTT<br>GTTCATTGATACGTTGGAGAGGGTATGGAAGAAGGAGAGTTTAGCGAGGCACG

	CGAAGATTTAGCGGCGTTAGAAAAGGATTATGAGGAAGTTGGTGTAGATTCTT
	TGGAGGATAATGGAGAAGAGGGAGATGAGTATTAA
Hcotbb-1 C-Strep tag II	ATGCGCGAAATTGTGCACGTGCAGGCGGGACAATGCGGCAACCAGATCGGCTC
	CAAGTTTTGGGAGGTGATCTCGGACGAGCACGGCATCCAACCAGACGGTACGT
	ACAAAGGCGAGAGCGATCTGCAGCTGGAGCGCATCAATGTGTACTACAATGAG
	GCGCACGGAGGCAAGTACGTGCCGCGCGCCGTGCTGGTGGACCTGGAACCGGG
	CACCATGGACAGCGTTCGTTCGGGTCCATACGGCCAGCTGTTCAGACCCGACA
	ACTACGTGTTTGGCCAGAGTGGCGCAGGCAATAACTGGGCGAAGGGCCACTAT
	ACAGAGGGCGCGGAGCTGGTCGACAACGTGCTGGACGTGGTGCGCAAGGAGGC
	CGAAGGCTGCGACTGTCTGCAGGGTTTCCAGCTGACCCACTCACTGGGTGGTG
	GCACTGGCTCTGGCATGGGCACCCTGCTGATCAGCAAGATTAGAGAAGAGTAC
	CCCGATAGGATCATGGCATCCTTCTCCGTGGTGCCAAGCCCCAAGGTCTCCGA
	CACCGTGGTTGAACCGTACAACGCCACGCTGTCGGTACACCAGCTGGTCGAGA
	ACACGGACGAGACTTTTTGCATCGACAACGAGGCTCTGTACGACATCTGCTTC
	CGCACGCTCAAGCTGACTAACCCTACATATGGCGACCTCAATCACCTGGTGAG
	CGTGACGATGAGCGGCGTCACGACCTGTCTTCGCTTCCCAGGCCAGCTGAACG
	CCGACCTGCGCAAGCTGGCTGTCAATATGGTTCCGTTCCCGCGGCTCCATTTC
	TTCATGCCAGGTTTCGCGCCCCTTTCCGCTAAGGGTGCACAAGCTTACCGCGC
	CTCCACAGTGGCCGAGCTGACCCAGCAGATGTTCGACGCAAAGAATATGATGG
	CAGCGTGCGACCCGCGTCACGGGCGCTACCTGACTGTCGCCGCCATGTTTCGC
	GGCCGTATGAGCATGCGGGAGGTGGACGACCAGATGATGTCTGTCCAGAACAA
	AAACTCGTCTTATTTTGTTGAATGGATCCCAAACAACGTTAAGACGGCGGTGT
	GCGACATACCGCCGCGGGGCCTCAAGATGGCAGCGACCTTCGTTGGCAACTCC
	ACGGCGATCCAGGAACTGTTCAAGCGGATCAGTGAGCAATTTACCGCTATGTT
	TCGGCGCAAGGCTTTCCTGCACTGGTACACGGGAGAAGGTATGGACGAGATGG
	AATTTACAGAGGCGGAAAGTAACATGAACGACCTTATCAGCGAGTATCAGCAA
	TATCAGGAGGCCACGGCAGATGACATGGGCGACCTGGACGCGGAAGGTGGAGA
	AGAACCCTACCCCGAAGAG**GAGAACCTTTACTTCCAGGGC*GGTGGCTCCGGCG***
	GCTGGTCACACCCCCAGTTTGAAAAGTGA
*Hcotbb-1** C-Strep tag II	**ATG**AGGGAGATTGTACATGTGCAGGCGGGTCAATGTGGAAACCAGATTGGCTC
	CAAGTTTTGAGAGGTAATTAGCGATGAGCATGGCATTCAACCCGACGGGACGT
	ACAAAGGAGAGAGCGATTTACAGCTGGAGCGTATAAATGTGTATTACAATGAA
	GCCCACGGGGGTAAATATGTACCACGGGCGGTGCTGGTAGATTTAGAACCCGG
	CACAATGGACAGTGTACGTAGTGGACCCTATGGACAATTATTTCGCCCGGATA
	ATTACGTTTTTGGTCAATCAGGTGCCGGAAATAATTGAGCAAAAGGCCACTAT
	ACCGAAGGTGCCGAGTTAGTGGATAACGTATTAGATGTGGTTCGTAAAGAAGC
	AGAGGGTTGCGACTGTTTGCAGGGATTTCAATTAACGCATTCTCTGGGTGGCG
	GTACCGGTTCAGGGATGGGTACATTATTAATTTCAAAGATAAGAGAGGAATAT
	CCAGACAGAATAATGGCATCGTTTAGCGTGGTACCTAGCCCAAAAGTTTCCGA
	TACTGTGGTTGAACCCTATAATGCAACACTGTCGGTACACCAGTTAGTCGAAA
	ACACGGATGAAACTTTTTGTATAGATAATGAGGCCTTATATGATATTTGTTTT
	AGAACATTAAAATTAACTAACCCGACATATGGAGATCTTAACCACCTTGTAAG
	CGTTACGATGAGTGGAGTAACAACATGTTTAAGATTTCCCGGACAATTAAATG
	CCGACTTAAGAAAATTAGCAGTGAATATGGTTCCGTTTCCTCGATTGCATTTT
	TTTATGCCTGGGTTTGCACCGTTATCGGCCAAAGGTGCTCAGGCATATCGTGC
	ATCCACGGTTGCAGAGTTAACGCAACAGATGTTTGACGCGAAAAATATGATGG
	CCGCGTGTGACCCCCGTCACGGGCGTTATTTAACAGTGGCGGCTATGTTTAGA
	GGACGCATGAGTATGCGGGAAGTTGACGATCAGATGATGAGTGTGCAGAATAA
	GAATTCTAGTTATTTTGTTGAGTGAATTCCTAATAACGTAAAGACGGCGGTTT
	GTGATATACCCCCGCGTGGCTTAAAAATGGCGGCTACATTTGTTGGAAACAGT
	ACGGCAATACAAGAGCTTTTTAAGAGAATATCAGAGCAATTTACAGCAATGTT
	TAGACGTAAAGCCTTTTTACACTGATATACAGGTGAGGGCATGGATGAGATGG
	AGTTTACTGAGGCGGAAAGCAATATGAATGATTTAATAAGTGAGTATCAGCAG
	TATCAAGAAGCAACAGCCGATGATATGGGTGATTTAGACGCCGAGGGAGGTGA
	GGAACCATACCCCGAAGAG**GAAAACTTATATTTTCAGGGA*GGAGGTAGCGGAG***
	GCTGATCTCACCCGCAATTCGAGAAGTAA

S2 Table. Primer sequences used in this study. The complementary ends for the HiFi DNA Assembly are shown in bold letters.

Primer	Sequence (5'->3')
Fwd_*Hco_tba-1*_codon_opt.*	**CTTGCTGTGCCTTGCCACCA**ATGCACCACCATCATCATC
Rev_*Hco_tba-1*_codon_opt.*	**GTGGTGATGGTGGTGGGTAC**TTAATACTCATCTCCCTCTTC
Fwd_*Hco_tbb-1*_codon_opt.*	**CTTGCTGTGCCTTGCCACCA**ATGAGGGAGATTGTACATG
Rev_*Hco_tbb-1*_codon_opt.*	**GTGGTGATGGTGGTGGGTAC**TTACTTCTCGAATTGCGG
Fwd_*Hco_tba-1*_codon_opt.	**CTTGCTGTGCCTTGCCACCA**ATGCATCATCACCATCAC
Rev_*Hco_tba-1*_codon_opt.	**GTGGTGATGGTGGTGGGTAC**TTAATACTCATCCCCCTC
Fwd_*Hco_tbb-1*_codon_opt.	**CTTGCTGTGCCTTGCCACCA**ATGCGCGAAATTGTGCAC
Rev_*Hco_tbb-1*_codon_opt.	**GTGGTGATGGTGGTGGGTAC**TCACTTTTCAAACTGGGGG
F3001	GATCTGGTTGATTCTGCCAGTAG
A1715	TATTCGTTGTCAGATGGCGCAC
P1442	CCGACTGCAACAAGGTGTAG
A264	CATCTATAGAGAAGTACACGTAAAAG
A3804	CCGATGGCTGTGTAGAAGTACTCG
D2999	CCTAGTATGAAGATTTCGGTGATC
F3002	CTGCAGGTTCACCTACAGCTAC
Fwd_*Hco_tbb-1**_RT	ATGAGCATGGCATTCAACCC
Rev_*Hco_tbb-1**_RT	CGAATTGCGGGTGAGATCAG
Fwd_*Hco_tbb-1*_RT	GTACTACAATGAGGCGCACG
Rev_*Hco_tbb-1*_RT	GGGTAGGGTTCTTCTCCACC

S3 Table. Optical density measurements at 600 nm of LEXSY cultures after Amaxa electroporation. Electroporation was performed on day –1 using the Amaxa system. On day 0, selection was initiated by the addition of 100 µg/mL nourseothricin and 100 µg/mL hygromycin. Values below the detection limit of 0.1 were marked as N/A.

	Day																		
	-1	0	1	2	3	4	5	6	7	8	9	10	11	12	13	14	15	16	17
Scrambled ORF1	0.233	0.521	0.771	1.026	1.274	1.553	1.603	1.692	1.877	1.890	1.622	1.290	0.814	0.591	0.473	0.357	0.681	1.497	3.010
Scrambled ORF2	0.233	0.445	0.710	0.994	1.172	1.431	1.511	1.589	1.753	1.721	1.499	1.111	0.699	0.476	0.364	0.252	0.483	1.369	2.870
Scrambled ORF3	0.233	0.427	0.663	0.890	1.118	1.411	1.499	1.553	1.765	1.717	1.481	1.098	0.672	0.456	0.358	0.247	0.462	1.363	2.775
Intact ORF1	0.233	0.431	0.665	0.928	1.063	1.459	1.509	1.556	1.477	1.389	1.239	0.894	0.672	0.467	0.330	0.166	N/A	N/A	N/A
Intact ORF2	0.233	0.529	0.781	1.002	1.189	1.603	1.674	1.705	1.591	1.322	1.322	1.098	0.744	0.559	0.391	0.214	N/A	N/A	N/A
Intact ORF3	0.233	0.438	0.659	0.935	1.042	1.420	1.544	1.531	1.468	1.189	1.189	0.903	0.690	0.404	0.311	0.139	N/A	N/A	N/A
Mock transfection1	0.233	0.389	0.687	0.915	1.013	1.414	1.522	1.579	1.615	1.659	1.301	0.854	0.642	0.427	0.297	0.110	N/A	N/A	N/A
Mock transfection2	0.233	0.371	0.691	0.998	1.134	1.603	1.653	1.555	1.591	1.513	1.394	1.031	0.704	0.540	0.359	0.191	N/A	N/A	N/A
Mock transfection3	0.233	0.382	0.713	0.924	1.008	1.451	1.530	1.588	1.602	1.483	1.267	0.833	0.650	0.366	0.288	0.149	N/A	N/A	N/A

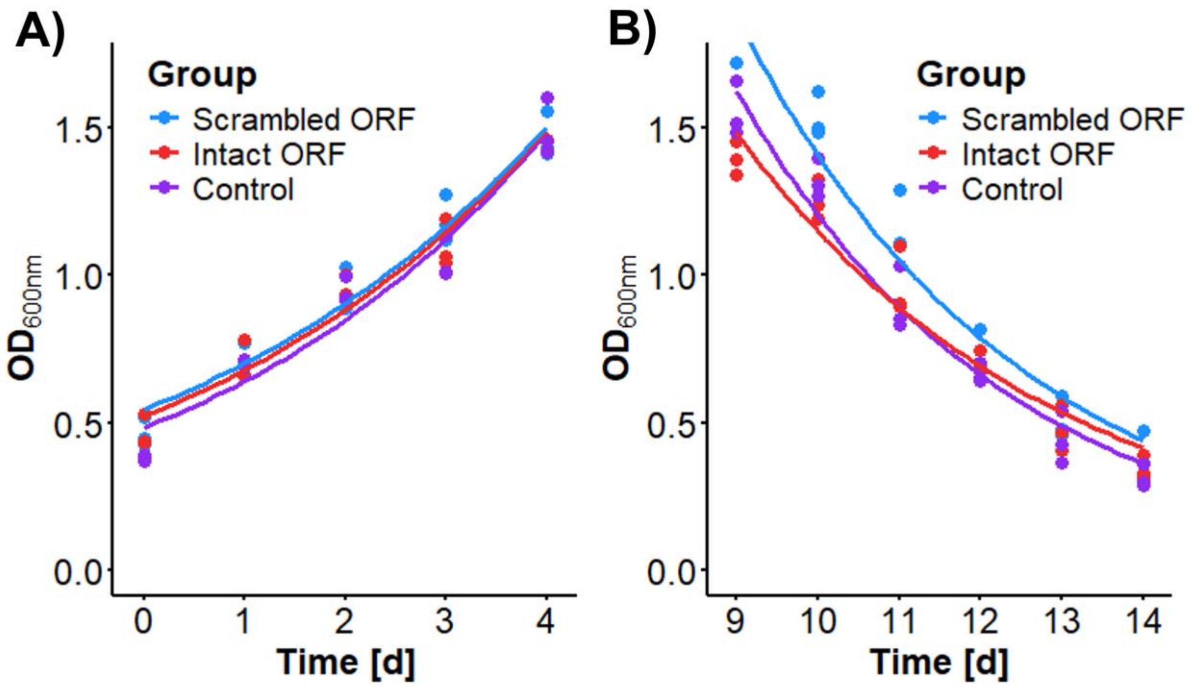

S1 Fig. Growth kinetics of transgenic LEXSY P10 in BHI+ medium at 26 °C. A) shows the lag phase, while B) shows the death phase of the selection curve. The exponential growth or decrease was described by the following formula: $y = y0 \times e^{(kx)}$. The doubling time was determined using the rate constant k as follows: $\ln(2) \times k^{-1}$.

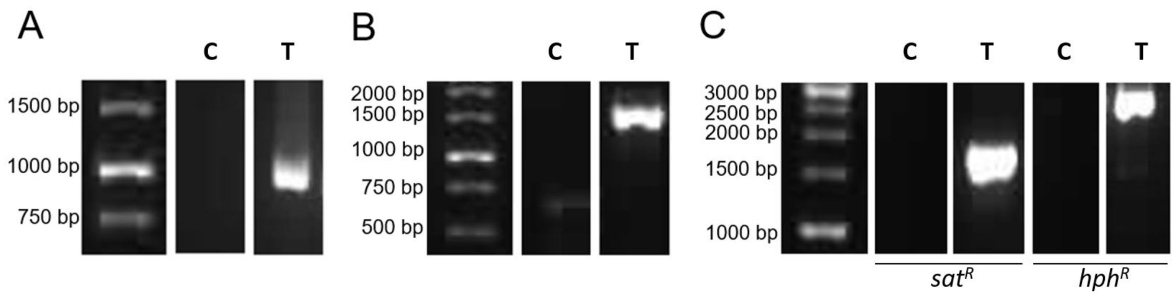

S2 Fig. Conventional PCRs were conducted to confirm the genomic integration. The gel images were edited to remove redundant lanes and improve the clarity and focus of the figure, while maintaining the integrity of the data presented. Lane C and Lane T correspond to the LEXSY host and LEXSY transgenic, respectively. A) PCR to confirm the successful integration of the expression cassettes at the left homology arm. B) PCR to confirm *Hcotba-1** and *Hcotbb-1**, both with scrambled ORFs. C) PCR to confirm the selection markers sat^R and hph^R.

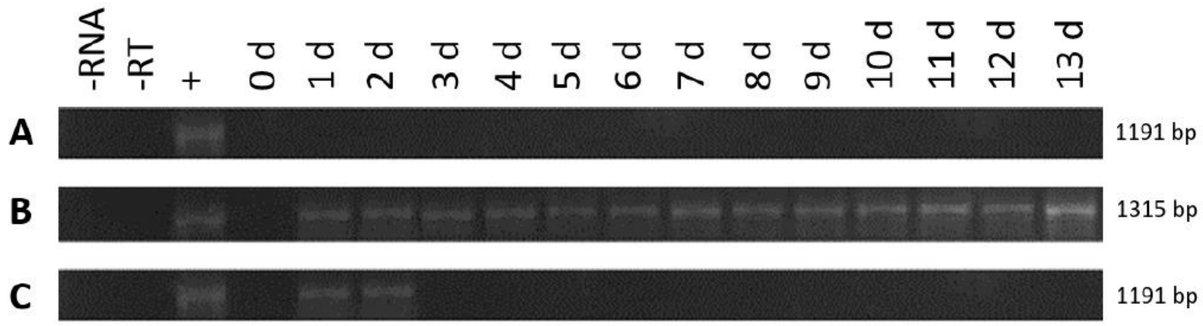

S3 Fig. RT-PCRs were performed to confirm the RNA expression of *Hcotbb-1*. A) mock transfection of LEXSY host promastigotes. B) LEXSY host promastigotes transfected with *Hcotba-1** and *Hcotbb-1**, both with scrambled ORFs. C) LEXSY host promastigotes transfected with *Hcotba-1* and *Hcotbb-1*, both with intact ORFs. Samples were collected on days (d) 0 ï 13 after the addition of antibiotics. -RNA, control without RNA added. -RT, control without reverse transcriptase added. +, Positive control consisting of gBlock Gene Fragment of *Hcotbb-1* and *Hcotbb-1**.
